# Antioxidant Activity and Polyphenols Characterization of Four Monovarietal Grape Pomaces from Salento (Apulia, Italy)

**DOI:** 10.3390/antiox10091406

**Published:** 2021-09-01

**Authors:** Carmine Negro, Alessio Aprile, Andrea Luvisi, Luigi De Bellis, Antonio Miceli

**Affiliations:** Department of Biological and Environmental Sciences and Technologies (DiSTeBA), Salento University, 73100 Lecce, Italy; alessio.aprile@unisalento.it (A.A.); andrea.luvisi@unisalento.it (A.L.); luigi.debellis@unisalento.it (L.D.B.); antonio.miceli@unisalento.it (A.M.)

**Keywords:** grape marc, polyphenols, antioxidant activity, wine by-product

## Abstract

The wine industry annually produces millions of tons of by-products rich in polyphenolic compounds that can be reused as secondary raw material in the food, cosmetic and pharmaceutical industries. The purpose of this work was to describe the presence of nutraceutical compounds and to evaluate the antioxidant activity of pomaces from three Apulian (South Italy, Italy) grape varieties (Negroamaro, Malvasia di Lecce and Primitivo) and to compare them with one of the most cultivated wines in Europe (Cabernet Sauvignon). The main classes of polyphenolic substances were characterized via high performance liquid chromatography/diode array detector/mass spectrometer time of flight (HPLC/DAD/TOF) and the antioxidant activity was evaluated with three different methods. The four investigated grape marcs have shown different polyphenols and antioxidant activities. Primitivo marc showed the higher antioxidant activity due to the excellent level of polyphenols, followed by the Negroamaro cultivar. In addition, marcs from traditional Apulian vines showed higher antioxidant activities than Cabernet Sauvignon because of an elevated level of active polyphenolic substances such as catechin, epicatechin, quercetin and its derivatives.

## 1. Introduction

It is well known that the agri-food industry by-products are rich in molecules with high nutraceutical values representing an excellent source of low-cost compounds to be employed as dietary supplements. Recently, public opinion has perceived dietary supplements as a “needed” nutritional and well-being element.

The wine growing and winemaking industry by-products represent a great opportunity. About 13 million tons [1] of waste materials are produced every year, which are rich in nutraceutical substances and complex carbohydrates; moreover, without their recycling, this biomass represents an environmental issue due to a high concentration of polyphenolic compounds and an elevated chemical oxygen demand (COD) [1]. The principal by-products in viticulture are the grape marc, which consists of grape stalks, seeds and skins left after the crushing and pressing stages of wine production, and lees.

The polyphenols mainly present in the grape marc are phenolic acids and flavonoids. Among the first the most abundant are derivatives of hydroxycinnamic acid, while within the second we identified several compounds belonging to diverse flavonoid subclasses, including anthocyanins, present in abundance and with different aglycones, flavan-3-oils, also represented by varying aglycones and degrees of polymerization, flavones and flavonols. [2]. Resveratrol is well-known for its nutraceutical properties [2], and it is also easily traceable in the grape marc. All these compounds have high antioxidant activities, and some of them have anti-inflammatory activities, too. Anthocyanins, moreover, could have anti-glycemic and anticancer effects, at least in vitro [3,4,5,6,7].

Of all the chemical classes present in the grape marc, the most abundant ones are the flavonoid family. The skins are the source of anthocyanins and flavonols, while in the seeds, the predominant compounds are flavan-3-ols, such as catechins and proanthocyanidins [2].

The grape marc characteristics are genotype-specific; anyway, they diverge significantly according to the growth area and climate conditions that greatly influence the presence of many chemical compounds [8]. For example, in Cabernet Sauvignon, a considerable qualitative/quantitative variation of anthocyanins was observed in response to temperature and water availability [9].

Therefore, the high diversity of vines grown in different regions limits the studies to the most common cultivars so that the available results are incomplete. Therefore, a characterization of unusual or rare varieties is required to support the reuse of marc in the context of agricultural biodiversity. The purpose of this work was, therefore, to describe the nutraceutical compounds and to evaluate the antioxidant activity of pomaces deriving from three Apulian (South-Italy) grape varieties (Negroamaro, Malvasia di Lecce and Primitivo) and to compare them with that of Cabernet Sauvignon, one of the most cultivated wines in Europe.

## 2. Materials and Methods

Industrial pomace of the varieties Negramaro, Malvasia di Lecce, Primitivo and C. Sauvignon vinified in purity by were analyzed to characterize their phenolic metabolites. All these grapes were cultivated in the Salento area (San Pietro Vernotico, province of Brindisi, Apulia, South Italy, Italy). The grapes came from the 15–20 year old vines, they were harvested in full maturity (at the beginning of September for Primitivo and at the end of September for the other three varieties) with entirely black/purplish berries during the 2019 vintage. The climate of the production area is temperate; the annual average temperature is 17.4 °C, the maximum 32 °C and the minimum 6.2 °C. The annual average rainfall is 628 mm. For all cv the berries are medium sized spheroids (13–17 mm), the bunches are tight, simple or sometimes winged or double; average bunch weight is approximately 200 g for Negroamaro and Malvasia di Lecce, 260 g for Primitivo and 120 g for C. Sauvignon.

Five grams (FW) of finely ground sample (taken from 500 g of finely ground grape marc resulting from the vinification of each of the four varieties) were extracted at room temperature with 100 mL of methanol 80% acidified with formic acid 0.1% for one hour in an ultrasonic bath. The extract was centrifuged, and the extraction was repeated on the pellet. The supernatants were mixed and evaporated, then resuspended with 25 mL of distilled water acidified with formic acid 0.1%.

The extract was purified by solid-phase extraction (SPE) Phenomenex Strata X columns (Phenomenex Italia, Castel Maggiore, Bologna, Italy) as previously reported [10]. After activation of the SPE cartridge with 2 mL of pure methanol and 5 mL of bi-distilled water, 25 mL of raw extract was loaded and washed with 25 mL of acidified (with 0.1% formic acid) bi-distilled water. Finally, 25 mL of acidified methanol (with 0.1% formic acid) was used to recover polyphenols compounds.

The purified extract was dried under vacuum and resuspended with high performance liquid chromatography (HPLC) water acidified with 0.1% formic acid. However, the high performance liquid chromatography/diode array detector/mass spectrometer time of flight (HPLC/DAD/TOF) analyses were carried out on the raw extract to characterize the chemical compounds present in the grape marc.

The total phenolic content (TPC) was determined using the spectrophotometric Folin–Ciocalteau method [10], the absorbance was measured with a JASCO V-550 UV/VIS spectrophotometer at 765 nm and data were expressed as gallic acid equivalent (GAE)·per mg/g dry weight (DW). Total anthocyanins (TA) were evaluated as reported by Di Stefano et al. [11], the absorbance at 520 nm was read with a JASCO V-550 UV/VIS spectrophotometer, and results were expressed as malvidin 3-*O*-glucoside equivalent (ME). The proanthocyanidin (PA) quantification was carried out after hydrolysis into cyanidins (in HCl 12 N plus 300 mg/L of FeSO_4_·7H_2_O for 50 min in a thermostatic bath at 100 °C, with reflux) at 520 nm. The results were expressed as mg/g DW cyanidin equivalent (CE) [11]. The total flavonoid content (TFC) was determined as indicated by Mitrevska et al. [12] using the spectrophotometric method based on NaNO_2_-AlCO_3_ reagent and data expressed as catechin equivalent (CaE)·mg/g DW.

Phenolic characterization was performed by an Agilent 1200 Liquid Chromatography system (Agilent Technologies, Palo Alto, CA, USA) equipped with a standard autosampler. The HPLC column was an Agilent Extended C18 (1.8 μm, 2.1 × 50 mm). Separation was carried out at 40 °C with a gradient elution program at a flowrate of 0.5 mL/min. The mobile phases consisted of water plus 0.1% formic acid (A) and acetonitrile (B). The following multistep linear gradient was applied: 0 min, 5% B; 13 min, 25% B; 19 min, 40% B. The injection volume in the HPLC system was 5 µL. The HPLC system was coupled to a DAD (Agilent Technologies, Palo Alto, CA, USA) set at 280 nm and an Agilent 6320 TOF mass spectrometer equipped with a dual electrospray ionization (ESI) interface (Agilent Technologies, Palo Alto, CA, USA) operating in negative ion mode. Detection was carried out within a mass range of 50–1700 *m*/*z*. Accurate measurements of the mass corresponding to each total ionic current (TIC) peak were obtained with a pump (Agilent G1310B) introducing a low flow (20 μL/min) of a calibration solution containing internal reference masses at *m*/*z* 112.9856, 301.9981, 601.9790, 1033.9881, and using a dual nebulizer ESI source in negative ion mode [13].

The anthocyanins were identified with the same chromatography system. Phase A was water plus 1% formic acid, and phase B was acetonitrile plus 1% formic acid. The HPLC column was an Agilent Extended C18 (1.8 μm, 2.1 × 50 mm). Separation was carried out at 40 °C with a gradient elution program at a 0.5 mL/min flow rate. The following multistep linear gradient was applied: 0 min, 5% B; 12 min, 15% B; 20 min, 30% B; 35 min. 45% B. The injection volume in the HPLC system was 5 µL. TOF operated with positive ionization, using the internal reference masses at *m*/*z* 121.0508, 149.0233, 322.0481 and 922.0097. Finally, wavelength DAD detection was 520 nm.

For both phenolic and anthocyanins, characterization mass spectrometer conditions were as follows: capillary voltage 3.0 kV in negative mode and 3.5 kV in positive mode; nitrogen was used as the nebulizer and desolvation gas; drying gas temperature: 300 °C; drying gas flow: 12 L/min, nebulizing gas pressure: 40 psig; finally, the source temperature was 120 °C. Mass Hunter software (Agilent Technologies, Palo Alto, CA, USA) was used to process the mass data of the molecular ions.

The compounds were quantified using calibration curves of authentic standards (gallic acid, caffeic acid, cumaric acid, catechin, epicatechin, quercetin 3-*O*-glucoside, quercetin, kaempferol, kaempferol 3-*O*-glucoside, resveratrol, cyanidin 3-*O*-glucoside, malvidin 3-*O*-glucoside, delphinidin 3-*O*-glucoside, petunidin 3-*O*-glucoside, peonidin 3-*O*-glucoside) purchased from Merck Life Science, Milano, Italy.

The evaluation of the antioxidant activity was carried out by testing three aspects: scavenger, reducing and quenching capacity.

DPPH Assay. Antioxidant activity was determined in vitro by evaluation of the free radical scavenging activity using 2,2-diphenyl-1-picrylhydrazyl (DPPH^•^) (DPPH assay) [14]. Inhibition of free radical DPPH^•^ was expressed as Trolox (6-hydroxy-2,5,7,8-tetra-methylchroman-2-carboxylic acid) equivalents (TE) per g DW.

Ferric Reducing Antioxidant Power (FRAP). The ferric reducing ability was determined by the FRAP method [15]. The absorption of the reaction mixture was measured at 593 nm using Perkin Elmer 2030 Multilabel reader Victor X5 after 3 min of incubation at 37 °C. The samples were measured in triplicate, and the FRAP was expressed as Trolox equivalents (TE)/g DW.

Superoxide anion scavenging activity assay. The assay was carried out according to Beauchamp and Fridovich [16]. The photo-induced reactions were performed using fluorescent lamps (200 W at 1 m). All samples were measured in triplicate, and the superoxide anion scavenging activity was expressed as IC_50_ μg/mL (IC = half maximal inhibitory concentration).

The dry weight (DW) of the marcs was determined at 105 °C until constant weight using a thermo-ventilated oven. The following equation was used for calculation: DW (%) = (W1 × 100)/W2 where W1 is the weight after drying and W2 is the weight of the original sample.

The yield was calculated from the following equation: Yield (%) = (W1 × 100)/W2 where W1 is the weight of the extract residue obtained after solvent removal and W2 is the weight of sample.

All data were reported as the mean ± standard deviation (SD), with at least three replications for each sample. Statistical evaluation was conducted by Duncan’s test to discriminate among the mean values. Pearson’s correlation was calculated to assess the correlation between antioxidant activity and individual compounds. All statistical analyses were performed using the software Statistica (StatSoft, Tulsa, OK, USA).

## 3. Results

### 3.1. Spectrophotometer and Gravimeter Determinations

The extraction efficiency (yield %) were similar for the four varieties with an average value close to 9%; in particular, as reported in Table 1, the values vary from 8.4% (Cabernet Sauvignon) to 9.2% (Primitivo). The water content was approximately 9%. Concerning the main polyphenolic classes in the grape marcs (Table 1) Negramaro showed the highest values, such as TPC content (52.9 mg/g DW), total flavonoids (38.4 mg/g) and total anthocyanins (10.3 mg/g). On the contrary, the Primitivo cv. showed the highest quantity of proanthocyanidins (2.1 mg/g). Moreover, Primitivo cv. reported high levels of TPC equal to 42.1 mg/g, as well as the content of total flavonoids and total anthocyanins, equal to 33.7 and 9.7 mg/g, respectively.

### 3.2. Characterization of Anthocyanins

**Figure 1:**
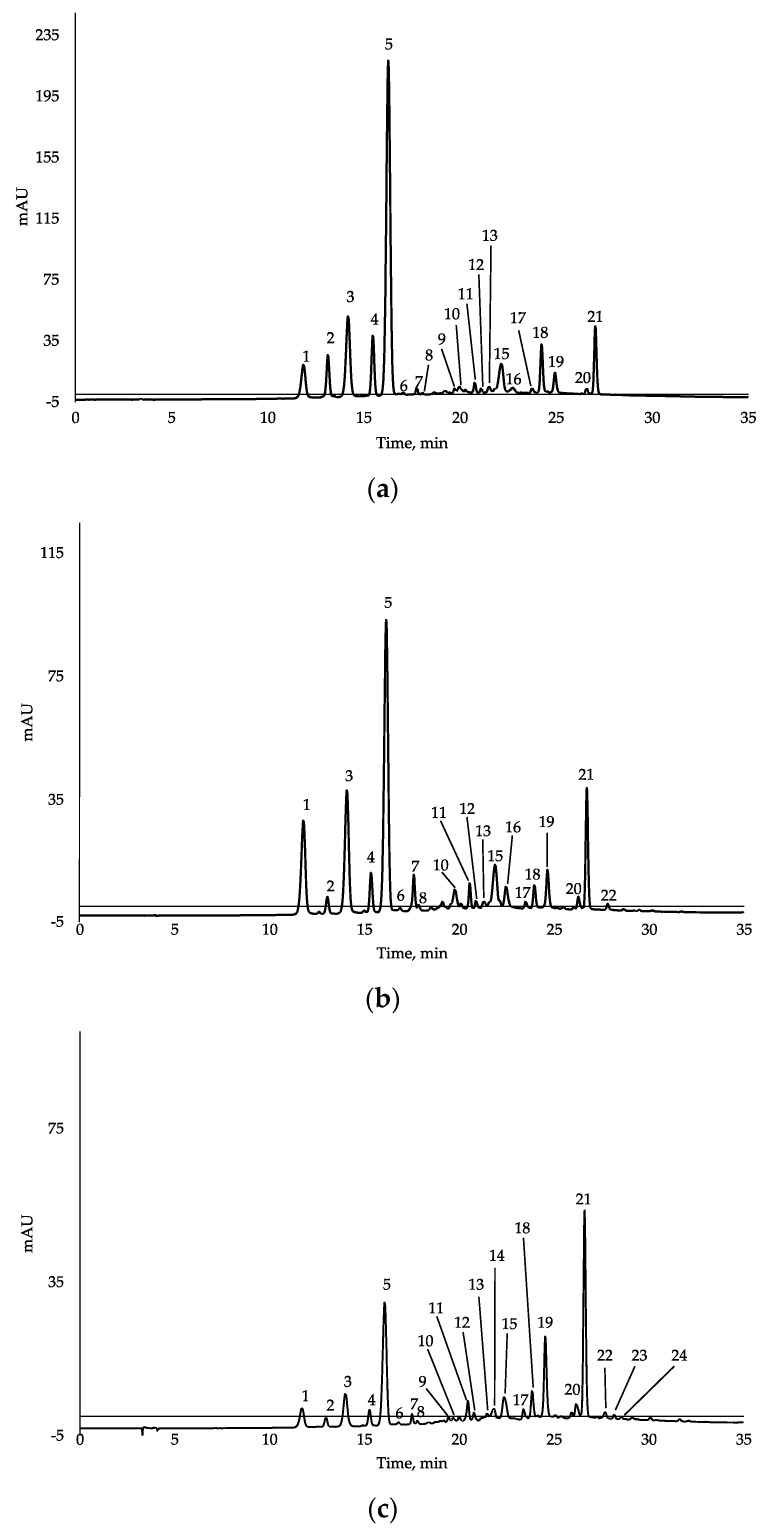

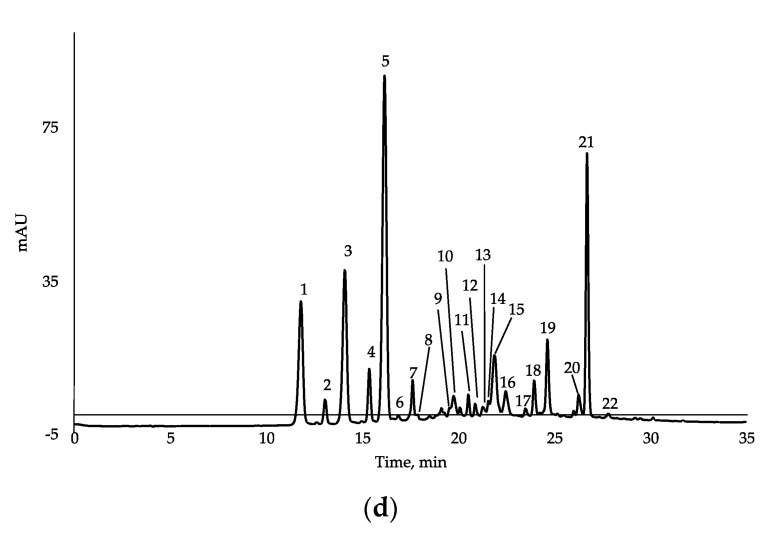
HPLC/DAD chromatogram (λ = 520 nm) of anthocyanins in four grape marcs. (**a**) Negroamaro; (**b**) Malvasia di Lecce; (**c**) Primitivo; (**d**) Cabernet Sauvignon. For identification of compounds see Table 2.

The qualitative characterization of the anthocyanins is listed in Table 2, while the UV/VIS chromatograms recorded at 520 nm are shown in Figure 1. A total of 24 compounds relative to five different aglycones were found. In particular, derivatives of cyanidin, malvidin, peonidin, delphinidin and petunidin were identified. We found typical grape cyanins that are known to be created during the fermentation process or during the ageing of wines, such as vitisin A and B. These compounds are the result of reactions between malvidin and pyruvic acid or acetic aldehyde, usually produced during the fermentation process [17].

These reactions involve other anthocyanins, too. Pyranopeonidin 3-*O*-glucoside was also observed, as well as carboxypyrano peonidin 3-*O*-glucoside and numerous derivatives of malvidin: malvidin 3-*O*-glucoside ethyl (epi)-catechin, pyranomalvidin 3-(6′coumaroyl)-glucoside, malvidin 3 Glucoside 4 vinylphenol, malvidin 3 glucoside 4 vinylsyringol, malvidin 3 glucoside 4 vinylguaiacol [20].

The quantitative analysis, expressed as µg/g DW, is displayed in Table 3; the most concentrated compound was the malvidin 3-*O*-glucoside. As observed, compounds vary according to the grape cultivar. In detail, the content of malvidin 3-glucoside ranged from 56 µg/g in Primitivo to 382 µg/g in Negroamaro marc which also contains the higher amount of petunidin 3-*O*-glucoside. A similar quantity of malvidin coumaroyl glucoside is present in all marcs, with Cabernet Sauvignon showing the higher amount, 77 µg/g. Instead, the most abundant compounds in Malvasia di Lecce marc are delphinidin 3-*O*-glucoside and malvidin acetyl glucoside.

### 3.3. Characterization of Other Substances

The HPLC/MS/TOF analysis in negative ion mode revealed the presence of 67 compounds (Table 4) of which 58 were identified: organic acids, hydroxybenzoic acids, hydroxycinnamic acids, flavonoids (flavonols, flavanols, flavones, flavan-3-oils and tannins), stilbenes. Among the organic acids we identified gluconic, galacturonic, pyruvic, tartaric, malic, fumaric, lactic, furonic, citric and suberic acid. In addition, we found other compounds derived and/or combined with phenolic compounds. Two hydroxybenzoic acids such as gallic acid and syringic acid and their derivatives or glucosides were identified, too [8,22]. Caffeic, ferulic and coumaric acids belong to the class of hydroxycinnamic acids: all of them were found in grape marc, as well as their glycosylated derivatives or replaced with hydroxyl groups [8,23]

The flavonoid class was even more copious. Compounds belonging to different subclasses, precursors and polymers were identified. In particular, these compounds belong to the subclass of flavan-3-ols, such as catechin, epicatechin and related tannins, that are well-known in grapes [8,23,24,25]. Flavonol compounds are very numerous, too: myricetin 3-*O*-hexoside, quercetin 3-*O*-glucoside, quercetin 3-*O*-galactoside, quercetin 3-*O*-rhamnoside, kaempferol 3-*O*-glucoside, kaempferol 3-*O*-hexuroside [8,26,27], were identified. Among flavononols, we found dihydroquercetin 3-*O*-rhamnoside (astilbin) [28,29]. Finally, we found only one stilbenoid compound: resveratrol [8,27].

The quantification of the most representative polyphenolic compounds is shown in Table 5. The highest concentration values were observed for catechin and epicatechin, followed by the glucosides of quercetin and kaempferol. In particular, the catechin content ranged between 0.45 and 2.54 mg/g DW in Malvasia di Lecce and Primitivo, respectively. The epicatechin concentration was 1.95 mg/g in Primitivo grape marc, whereas it was 0.25 mg/g in Malvasia di Lecce. Regarding the flavonol compounds, the most abundant were the quercetin derivatives, in particular the quercetin glucuronide ranging from 0.05 (Primitivo) to 0.91 mg/g DW (Malvasia di Lecce). Negramaro grape marc showed a high concentration of quercetin glucoside (1.56 mg/g DW), while in the other cultivars we detected significantly lower quantities.

### 3.4. Determination of Antioxidant Activity

The antioxidant activity of the extracts obtained from the four grape marcs were evaluated using three different methodologies (Table 6). Regarding the DPPH test, the Primitivo grape marc showed the highest TEAC value (251 μmol TE/g DW). The other three cultivars showed lower TEAC values, ranging from 122 to 141 μmol TE/g DW. The FRAP test provided similar values: the best antioxidant activity values were observed in the Primitivo grape marc (127 μmol TE/g DW), while the other extracts showed lower or similar values. The superoxide anion test confirmed the previous results. The lowest IC_50_ value was observed in Primitivo grape marc, followed by Malvasia di Lecce, Negroamaro and Cabernet Sauvignon.

Table 7 reports the correlation between the results of the antioxidant activity tests and the polyphenolic compounds measured in the different samples. The data presented show that the DPPH and FRAP tests are in agreement and significantly correlated with the same compounds, i.e., with phenolic acids (gallic and caffeic), flavan3ols (catechin, epicatechin, galloylcatechin), flavonols and derivatives (quercetin, kaempferol). The superoxide anion test shows a similar correlation trend even if the Pearson’s correlation values are lower than DPPH and FRAP tests.

## 4. Discussion

TPC values of the four grape marcs closely match the data reported by several other authors [36,44,45] that have observed phenolic content levels between 30 and 70 mg/g DW. In a previous work focused on Negroamaro wine a similar TPC value (41.9 mg/g DW) was recorded [46]. However, the genotype is not the unique element that impacts the TPC in grape marc. In fact, the TPC values are influenced by the winemaking techniques, weather and growing region [47,48].

The anthocyanin aglycones are widely reported in the literature, while the derivatives can vary a lot from sample to sample. For instance, Oliviera et al. [17] in grape marc obtained from the main red grape varieties of the Douro Region (Portugal) identified 50 compounds with molecular weights ranging from 465 and 1623 Da after a MALDI TOF analysis. On the contrary, a Q-TOF analysis on Cabernet Sauvignon grape marc identified only eight compounds [36], and a different study on Sicilian samples identified 11 anthocyanin derivatives [49,50]. This qualitative/quantitative variation suggests a great intraspecific biodiversity that could also be used to identify grape varieties. In fact, the compound ratios seem to be specific for each cultivar [48].

From a nutraceutical and industrial point of view, the simultaneous presence of compounds deriving from berry metabolism and molecules modified by yeasts during fermentation make marc matrices very interesting; indeed, it has been suggested that the anthocyanins which have many substitute groups are more resistant to degradation during heating and at the same time are less affected by pH variations [51,52,53]. Additionally, the non-acylated anthocyanins have a more significant anti-inflammatory activity, at least in in vitro test [54]. However, further studies are needed to confirm these indications.

The data shown in this study fall within the averages of the values obtained from vines grown in similar conditions. For example, in pomace from Sicilian productions, the content of malvidin 3-*O*-glucoside ranged between 23 and 200 µg/g depending on the cultivar [49]. Different authors, instead [55,56], reported lower values confirming that the concentrations are influenced by agronomic input as well as genetic factors [8,47,48].

The organic acids identified derive both from the berry and the fermentation processes. In particular, among the organic acids, pyruvic and lactic acids are mainly produced during fermentation process, while tartaric and malic are produced in the berries [31], as well as galacturonic [30]. Whereas, gluconic acid can be an indicator of the presence of fungi in grapes [37,38].

Quercitin and kaempferol and their derivatives showed a high concentration in grape marc and many authors have already demonstrated their beneficial biological activities [57,58]. Among quercetin derivatives, astilbin is very interesting since it possesses antioxidant and anti-inflammatory activity, antirheumatic properties and it seems to have protective effects on the nervous system [59]. Astilbin probably comes from the stalks present in the grape marc [60], so that it is more easily recovered after fermentation. Indeed, it has been observed that the fermentation processes can increase the nutraceutical value due to the cell wall degradation by yeasts [61]. The astilbin content was lower than the other compounds observed in grape marc, but the values were similar to data reported by other authors [62] who found a great genotype influence on the astilbin values in a range from 3.75 to 7.57 µg/g DW.

The flavan-3-ol levels were similar to values reported in the literature, where catechin ranged from 0.94 to 1.50 mg/g [44]; in other conditions [55] the catechin amount was 0.5 mg/g, suggesting the role of climatic conditions and agronomic techniques in compound concentration [47]. Regarding quercetin and its derivatives, the values shown in previous works are not in agreement. For example, Bonilla et al. [55] reported 0.24 mg/g, Amico et al. [56] 0.32 mg/g; anyway, other authors observed lower levels of quercetin, ranging from 0.02 to 0.11 mg/g [2].

All tested extracts showed high antioxidant activities correlated with the content of gallic acid, flavan-3-ols (mainly catechin and epicatechin) and flavonols (quercetin and derivatives), compounds characterized by high antioxidant activity [63,64]. The anthocyanidin contents were less correlated to antioxidant activities, probably because they are present in lower amounts. Some studies have reported equivalent results; in particular, similar values were obtained in Argentina: a TEAC value of 150 and 73 μmol TE/g DW after DPPH and FRAP tests, respectively, for Cabernet Sauvignon grape marc [62]. In another work the mean value of TEAC was approximately 51 μmol TE/g DW (DPPH assay) [65].

The four investigated grape marcs showed different polyphenols and antioxidant activities. Primitivo marc showed a higher level of polyphenols, followed by Negramaro. All the traditional Apulian wines exhibit higher antioxidant activities than Cabernet Sauvignon as a consequence of a greater quantity of active polyphenolic substances such as catechin, epicatechin, quercetin and its derivatives.

## 5. Conclusions

In conclusion, the grape by-products represent a source of valuable ingredients for new foods, cosmetics and supplements being particularly rich in chemical compounds known for their valuable biological activities. Regardless, the presence of the different compounds varies in relation to the grape cultivar, so that only extracts from specific grape marc could have a beneficial and profitable use.

## Figures and Tables

**Table 1:** Content of total phenolic compounds (TPC, expressed as GAE), total flavonoid compounds (TFC, as CaE), total anthocyanins (TA, as ME), proanthocyanidins (PA, CE) reported as mg/g DW. Yield and moisture (%) in grape marc extracts are included.

	Sample			
	Negroamaro	Malvasia di Lecce	Primitivo	C. Sauvignon
TPC (mg/g DW)	52.9 ± 3.5 ^a^	27.6 ± 2.2 ^b^	42.1 ± 4.1 ^a^	38.1 ± 3.5 ^a^
TFC (mg/g DW)	38.4 ± 2.3 ^a^	20.7 ± 3.3 ^c^	33.7 ± 4.3 ^a^	27.6 ± 5.4 ^b^
TA (mg/g DW)	10.3 ± 1.4 ^a^	9.2 ± 1.4 ^a^	9.7 ± 2.1 ^a^	5.3 ± 1.7 ^b^
PA (mg/g DW)	1.7 ± 0.6 ^a^	1.4 ± 0.2 ^a^	2.1 ± 0.5 ^a^	1.2 ± 0.2 ^a^
Yield (%)	9.0 ± 0.5 ^a^	8.8 ± 0.7 ^a^	9.2 ± 0.5 ^a^	8.4 ± 0.6 ^a^
Moisture (%)	9.4 ± 0.5 ^a^	10.1 ± 0.5 ^a^	8.7 ± 0.5 ^a^	9.2 ± 0.5 ^a^

In the same row, different letters correspond to statistically different means (Duncan’s test, *n* = 3, *p* < 0.05).

**Table 2:** Anthocyanins [M-H]^+^ identification attempt by HPLC/DAD/TOF analysis in the grape marcs analyzed.

No.	Name	Formula [M-H]^+^	MW Exp [M-H]^+^	MW Calc [M-H]^+^	Δ ppm.	Score	Refs.
1	^1^ Delphinidin 3-*O*-glucoside	C_21_H_21_O_12_	465.1023	465.1033	−2.15	89	[18]
2	^1^ Cyanidin 3-*O*-glucoside	C_21_H_21_O_11_	449.1078	449.1089	−2.45	91	[18]
3	^1^ Petunidin 3-*O*-glucoside	C_22_H_23_O_12_	479.1181	479.1195	−2.92	92	[19]
4	^1^ Peonidin 3-*O*-glucoside	C_22_H_23_O_11_	463.1255	463.1245	2.16	89	[19]
5	^1^ Malvidin 3-*O*-glucoside	C_23_H_25_O_12_	493.1340	493.1346	−1.21	91	[19]
6	Pyrano Peonidin 3-*O*-glucoside	C_24_H_23_O_11_	487.1251	487.1244	−1.43	92	[19]
7	Carboxy pyrano cyanidin 3-*O*-glucoside	C_24_H_21_O_13_	517.0962	517.0987	4.83	91	[20]
8	Vitisin B	C_25_H_25_O_12_	517.1366	517.1351	2.90	89	[19]
9	Petunidin 3 acetyl-glucoside	C_24_H_25_O_13_	521.1286	521.1295	−1.72	93	[18,19]
10	Vitisin A	C_26_H_25_O_14_	561.1250	561.1244	−1.07	97	[19]
11	Carboxypyrano Delphinidin 3-*O*-glucoside	C_24_H_21_O_14_	533.0931	533.0936	−0.93	95	[19]
12	Malvidin 3-*O*-glucoside ethyl (epi-)catechin	C_40_H_41_O_18_	809.231	809.229	2.47	95	[20]
13	Peonidin 3 acetyl-glucoside	C_24_H_25_O_12_	505.1355	505.1346	1.78	89	[21]
14	Malvidin 3-*O*-glucoside ethyl (epi-)catechin	C_40_H_41_O_18_	809.230	809.229	1.23	91	[20]
15	Malvidin 3-(6′acetyl)-glucoside	C_25_H_27_O_13_	535.1421	535.1452	−5.79	87	[19,21]
16	Delphinidin 3 (6”coumaroyl)-glucoside	C_30_H_27_O_14_	611.1412	611.1406	0.98	89	[19]
17	Pyrano Malvidin 3-(6′coumaroyl)-glucoside	C_34_H_31_O_14_	663.1735	663.1719	2.41	91	[20]
18	Malvidin 3-(6′caffeoil)-glucoside	C_32_H_31_O_15_	655.1688	655.1668	−3.05	89	[20]
19	Petunidin 3-(6′coumaroyl)-glucoside	C_31_H_29_O_14_	625.1545	625.1557	−1.91	92	[19]
20	Peonidin 3-(6′coumaroyl)-glucoside	C_31_H_29_O_13_	609.1589	609.1608	−3.11	90	[19]
21	Malvidin 3-(6′coumaroyl)-glucoside	C_32_H_31_O_14_	639.1720	639.1714	−0.93	92	[19]
22	Malvidin 3-*O*-glucoside 4 vinylphenol	C_31_H_29_O_13_	609.1633	609.1613	3.28	91	[20]
23	Malvidin 3-*O*-glucoside 4 vinylsyringol	C_33_H_33_O_15_	669.1853	669.1813	5.97	89	[20]
24	Malvidin 3-*O*-glucoside 4 vinylguaiacol	C_32_H_31_O_14_	639.1731	639.1708	3.59	89	[20]

^1^ confirmed by standard compound.

**Table 3:** HPLC/MS quantification of the main anthocyanin compounds in the grape marcs (µg/g DW).

		Sample			
No.	Compound	Negroamaro	Malvasia di Lecce	Primitivo	C. Sauvignon
1	Delphinidin 3-*O*-glucoside	43 ± 2 ^b^	57 ± 2 ^a^	7 ± 1 ^c^	51 ± 2 ^a^
2	Cyanidin 3-*O*-glucoside	37 ± 3 ^a^	10 ± 1 ^b^	3 ± 1 ^c^	6 ± 3 ^b c^
3	Petunidin 3-*O*-glucoside	100 ± 3 ^a^	73 ± 2 ^b^	15 ± 3 ^d^	43 ± 2 ^c^
4	Peonidin 3-*O*-glucoside	55 ± 5 ^a^	17 ± 3 ^b^	5 ± 2 ^c^	8 ± 3 ^b c^
5	Malvidin 3-*O*-glucoside	382 ± 5 ^a^	167 ± 3 ^b^	56 ± 5 ^c^	158 ± 4 ^b^
15	^1^ Malvidin 3-(6′acetyl)-glucoside	3 ± 2 ^c^	38 ± 1 ^a^	19 ± 5 ^b^	35 ± 3 ^a^
19	^2^ Pet. 3-(6”coumaroyl)-glucoside	26 ± 2 ^a^	22 ± 6 ^a^	31 ± 3 ^a^	32 ± 3 ^a^
21	^1^ Mal. 3-(6”coumaroyl)-glucoside	50 ± 3 ^b^	45 ± 7 ^b^	51 ± 4 ^b^	77 ± 3 ^a^

^1^ determined as malvidin 3-*O*-glucoside, ^2^ determined as petunidin 3-*O*-glucoside. In the same row, different letters correspond to statistically different means (Duncan’s test, *n* = 3, *p* < 0.05).

**Table 4:** Compound [M-H]^−^ identification attempts by HPLC/DAD/TOF analysis in the grape marcs analyzed.

No.	Name	Formula [M-H]^−^	MW Exp [M-H]^−^	MW Calc [M-H]^−^	Δ ppm	Score	Refs.
1	^1^ Gluconic Acid	C_6_H_11_O_7_	195.0518	195.0510	−3.71	96.27	[20]
2	Galacturonic Acid	C_6_H_9_O_7_	193.0359	193.0354	−2.59	85.71	[20,30]
3	^1^ Pyruvic Acid	C_3_H_3_O_3_	87.0082	87.0088	7.02	85.02	[31]
4	^1^ Tartaric Acid	C_4_H_5_O_6_	149.0095	149.0092	−2.04	99.42	[20,31]
5	^1^ Gliceraldeide	C_3_H_3_O_2_	71.0127	71.0139	15.69	68.66	-
6	Trehalose	C_12_H_21_O_11_	341.1094	341.1089	−1.27	95.32	[20]
7	^1^ Malic Acid	C_4_H_5_O_5_	133.0143	133.0142	−0.72	87.3	[31]
8	^1^ Fumaric Acid	C_4_H_3_O_4_	115.0037	115.0037	0.18	86.93	[31]
9	^1^ Lactic Acid	C_3_H_5_O_3_	89.024	89.0244	5.21	85.36	-
10	Furonic Acid	C_5_H_3_O_3_	111.0087	111.088	0.42	87.72	[22]
11	^1^ Citric Acid	C_6_H_7_O_7_	191.0202	191.0197	−2.71	85.48	-
12	Unknown	C_5_H_5_O_4_	129.0194	129.0193	−0.42	87.56	-
13	Deoxy-D-Xylulose	C_5_H_9_O_4_	133.0512	133.0506	−4.08	97.15	[32]
14	Succinic Acid	C_4_H_5_O_4_	117.0195	117.0193	−0.68	87.22	[26,33]
15	^1^ Propionic Acid	C_3_H_5_O_2_	73.0287	73.0295	11.04	82.44	-
16	5-Hydroxymethyl-2-Furaldehyde	C_6_H_5_O_3_	125.025	125.0247	−2.91	96.2	[34]
17	4-Hydroxyphenylacetyl-Hexose	C_14_H_17_O_8_	313.091	313.0929	6.1	75.77	[33]
18	^1^ Gallic Acid	C_7_H_5_O_5_	169.0151	169.0142	−5.02	94.95	[8,22]
19	Dihydroxybenzoic Acid Hexoside	C_13_H_15_O_9_	315.0741	315.0722	−5.04	85.18	[8,35]
20	Gallic Acid Exoside	C_13_H_15_O_10_	331.0693	331.0671	−6.02	92.14	[8,35]
21	^1^ Caffeic Acid	C_9_H_7_O_4_	179.036	179.035	−5.86	93.56	[8,36]
22	Unknown	C_14_H_19_O_8_	315.110	315.1085	−3.64	88.97	-
23	Chalcan-Flavan-3-ol Dimer	C_30_H_27_O_12_	579.1519	579.1508	−1.09	90.11	[8]
24	Unknown	C_12_H_21_O_8_	293.1256	293.1242	−5.02	89.96	-
25	^1^ Catechin	C_15_H_13_O_6_	289.0748	289.0718	−9.18	73.72	[8,37,38]
26	(Epi)Catechin-(4,8″)-(Epi)Catechin	C_30_H_25_O_12_	577.1346	577.1351	1.56	92.36	[8,27]
27	(Epi)Catechin-(4,8″)-(Epi)Catechin	C_30_H_25_O_12_	577.1349	577.1351	0.73	68.57	[8,27]
28	Coumaric Acid Hexoside	C_15_H_17_O_8_	325.0947	325.0929	−3.89	86.39	[8]
29	Dihydrophaseic Acid Glucoside	C_21_H_31_O_10_	443.1948	443.1923	−4.73	73.97	[39]
30	Benzylalcohol Apiosylglucoside	C_19_H_27_O_12_	447.1518	447.1508	2.94	89.26	[34]
31	Cumaric Acid Hexoside Is II	C_15_H_17_O_8_	325.0948	325.0929	−3.89	83.78	[8]
32	^1^ Epicatechin	C_15_H_13_O_6_	289.0747	289.0718	−9.18	84.13	[8,27]
33	Gallic Acid Ethyl Ester	C_9_H_9_O_5_	197.0461	197.0455	−4.62	84.35	[8]
34	(Epi)Catechin-(4,8″)-(Epi)Catechin	C_30_H_25_O_12_	577.1344	577.1351	2.06	92.27	[8,27]
35	(Epi)Catechin-(4,8″)-(Epi)Catechin	C_30_H_25_O_12_	577.1350	577.1351	0.89	85.78	[8,27]
36	Malic Acid Derivative	C_20_H_31_O_10_	431.1937	431.1923	−2.89	87.76	[39,40]
37	Suberic Acid	C_8_H_13_O_4_	173.0829	173.0819	−5.42	93.23	[41]
38	4-Penten-1-yl α-D-Gluco-Pyranoside	C_11_H_19_O_6_	247.1203	247.1187	−5.05	88.17	
39	3-*O*-Galloyl(Epi)Catechin-(4,8″)-(Epi)Catechin	C_37_H_29_O_16_	729.1456	729.1461	1.17	83.19	[42]
40	Myricetin 3 Hexoside	C_21_H_19_O_13_	479.0843	479.089	4.89	89.79	[8,27]
41	Tetrahydroxy-Dimethoxyflavanone-Hexoside	C_23_H_25_O_13_	509.1322	509.1301	−3.3	79.4	[27]
42	Unknown	C_27_H_37_O_14_	585.2199	585.2129	−0.66	92.21	-
43	Unknown	C_10_H_17_O_3_	185.1204	185.1183	−5.57	77.44	-
44	Quercetin 3-*O*-(6″-Rhamnosyl)Hexoside	C_27_H_29_O_16_	609.1472	609.1461	−0.99	90	[8,27]
45	^1^ Quercetin 3-*O*-Glucoside	C_14_H_23_O_17_	463.0907	463.0941	6.1	73.65	[8,27]
46	^1^ Quercetin	C_15_H_9_O_7_	301.0351	301.0354	−3.34	93.23	[8,27,36]
47	Quercetin 3-*O*-Hexuronide	C_21_H_17_O_13_	477.0695	477.0675	−6.01	83.34	[8,36]
48	Quercetin 3-*O*-Hexoside	C_14_H_23_O_17_	463.0919	463.0941	6.09	85.06	[8,27]
49	Dihydroquercetin-3-*O*-Rhamnoside (Astilbin)	C_21_H_21_O_11_	449.1107	449.1089	−1.23	87.89	[28,29]
50	Larycitrin-3-*O*-Hexoside	C_22_H_21_O_13_	493.0999	493.0988	−1.91	91.56	[27]
51	Kaempferol-Dimethoxy Derivative	C_17_H_15_O_9_	363.074	363.0722	−3.89	84.53	[39]
52	Caffeoyl-Malic Acid	C_12_H_21_O_6_	261.1347	261.1344	−1.1	86.06	[43]
53	^1^ Kaempferol 3-*O*-Glucoside	C_21_H_19_O_11_	447.0949	447.0933	−3.13	85.6	[8,26,27]
54	Caffeoyl-Malic Acid Is. II	C_12_H_21_O_6_	261.1368	261.1344	−8.06	73.99	[28]
55	^1^ Sinapyl Alcohol	C_11_H_13_O_4_	209.0809	209.0819	5.53	88.81	-
56	Quercitin 3 Rhamnoside	C_21_H_19_O_11_	447.0962	447.0933	−5.46	77.84	[8]
57	Kaempferol 7-*O*-Hexuronide	C_21_H_17_O_12_	461.0747	461.0725	−3.76	82.76	[8]
58	2,4-Octadienoic Acid 7-Hydroxy-6-Methyl	C_9_H_13_O_3_	169.0881	169.087	−6.62	79.25	[8]
59	Resveratrol	C_14_H_11_O_3_	227.0717	227.0705	5.28	95.57	[8,27]
60	Syringetin 3-*O*-Hexoside	C_23_H_23_O_13_	507.1159	507.1144	−2.28	85.3	[8,27]
61	Butyl-Ethyl Succinate	C_10_H_17_O_4_	201.1146	201.1132	−6.09	88.47	[26]
62	Unknown	C_11_H_19_O_5_	231.1254	231.1238	−5.17	87.37	-
63	Unknown	C_15_H_11_O_5_	271.0616	271.0612	−1.56	95.57	-
64	^1^ Kaempferol	C_15_H_9_O_6_	285.0411	285.0405	−2.12	93.68	[8]
65	Unknown	C_18_H_33_O_5_	329.2352	329.2333	−4.99	82.26	-
66	Unknown	C_21_H_35_O_9_	431.2308	431.2287	−4.1	77.68	-
67	Unknown	C_11_H_13_O_3_	193.0881	193.087	−4.76	91.4	-

^1^ confirmed by the standard compound.

**Table 5:** HPLC/MS quantification of the main polyphenolic compounds in the grape marcs, reported as mg/g DW.

	Marc Sample			
Compound	Negroamaro	Malvasia di Lecce	Primitivo	C. Sauvignon
Gallic acid	1.03 ± 0.08 ^a^	0.51 ± 0.06 ^c^	1.76 ± 0.12 ^a^	0.73 ± 0.04 ^b^
Caffeic acid	1.21 ± 0.12 ^b^	1.05 ± 0.12 ^b^	1.81 ± 0.13 ^a^	1.03 ± 0.07 ^b^
^1^ Cumaric acid exoside	0.28 ± 0.02 ^b^	0.32 ± 0.10 ^b^	0.36 ± 0.02 ^b^	0.51 ± 0.02 ^a^
(±)Catechin	1.48 ± 0.05 ^b^	0.45 ± 0.09 ^c^	2.54 ± 0.13 ^a^	0.57 ± 0.06 ^c^
(±)Epicatechin	1.16 ± 0.03 ^b^	0.25 ± 0.06 ^d^	1.95 ± 0.11 ^a^	0.50 ± 0.05 ^c^
^2^ Galloyl(Epi)Catechin-(4,8″)-(Epi)Catechin	0.09 ± 0.03 ^a^	0.06 ± 0.02 ^b^	0.14 ± 0.03 ^a^	0.07 ± 0.04 ^a^
^3^ Quercetin glucuronide	0.20 ± 0.03 ^b^	0.91 ± 0.03 ^a^	0.05 ± 0.02 ^c^	0.07 ± 0.03 ^c^
Quercetin glucoside	1.56 ± 0.09 ^a^	0.03 ± 0.02 ^c^	1.37 ± 0.04 ^a^	0.13 ± 0.02 ^b^
Kampferol glucoside	0.14 ± 0.05 ^a^	<LOQ	0.04 ± 0.02 ^b^	<LOQ
^3^ Quercetin ramnoside	0.48 ± 0.02 ^a^	<LOQ	0.05 ± 0.01 ^b^	<LOQ
^3^ Astilbin (μg/g DW)	4.20 ± 0.12 ^a^	3.12 ± 0.06 ^b^	4.03 ± 0.13 ^a^	3.59 ± 0.09 ^b^
Quercetin	0.22 ± 0.03 ^b^	0.23 ± 0.06 ^b^	0.82 ± 0.03 ^a^	0.07 ± 0.03 ^c^
Kampferol	0.03 ± 0.01 ^a^	0.04 ± 0.02 ^a^	0.05 ± 0.03 ^a^	<LOQ
Resveratrol	0.10 ± 0.02 ^a^	0.08 ± 0.07 ^a^	0.12 ± 0.05 ^a^	0.09 ± 0.04 ^a^

^1^ determined as cumaric acid, ^2^ determined as catechin, ^3^ determined as quercetin glucoside. Different letters correspond to statistically different means (Duncan’s test, *n* = 3, *p* < 0.05). LOQ = limit of quantitation.

**Table 6:** Antioxidant activity tests (DPPH, FRAP and superoxide anion). The analyses were carried out on the SPE purified grape marcs. The results are reported as TEAC (μmol Trolox equivalents (TE)/g DW) for DPPH and FRAP test and in IC_50_ μg/mL for superoxide anion test. Different letters correspond to statistically different means (Duncan’s test, *n* = 3, *p* < 0.05).

	Marc Sample			
Test	Negroamaro	Malvasia di Lecce	Primitivo	C. Sauvignon
DPPH	141 ± 2 ^b^	122 ± 2 ^c^	251 ± 5 ^a^	126 ± 5 ^c^
FRAP	74 ± 7 ^b^	58 ± 6 ^c^	127 ± 3 ^a^	71 ± 4 ^b^
Superoxide anion	18 ± 3 ^c^	10 ± 1 ^b^	5 ± 2 ^a^	13 ± 4 ^b^

**Table 7:** Pearson correlation analysis between the polyphenolic compounds and the antioxidant activities among the four grape cultivars. The values marked in red show the highest correlation, those in green the lowest. Color legend: for values from 1.0 to 0.75 = red, from 0.75 to 0.50 = dark orange; from 0.50 to 0.25 = orange; from 0.25 to 0.0 = yellow; from 0.0 to −0.25 = yellowish; from −0.25 to −0.50 = light green; from −0.50 to −0.75 = green; from −0.75 to −1.0 = dark green.

Compound	DPPH	FRAP	Superoxide Anion
Gallic acid	0.96	0.98	0.50
Caffeic acid	0.99	0.98	0.66
Cumaric acid exoside	−0.11	−0.01	0.14
Catechin	0.94	0.94	0.42
Epicatechin	0.92	0.94	0.38
Galloyl-(Epi)Catechin	0.97	0.98	0.53
Quercetin glucuronide	−0.48	−0.61	0.04
Quercetin glucoside	0.97	0.97	0.51
Kaempferol glucoside	0.08	0.10	−0.60
Quercetin ramnoside	−0.11	−0.09	−0.73
Astilbin	0.52	0.59	−0.23
Quercetin	0.97	0.93	0.76
Kaempferol	0.62	0.50	0.54
Resveratrol	0.93	0.96	0.41
Delphinidin 3 glucoside	−0.99	−0.99	−0.61
Cyanidin 3 glucoside	−0.35	−0.35	−0.84
Petunidin 3 glucoside	−0.71	−0.73	−0.84
Peonidin 3 glucoside	−0.36	−0.36	−0.83
Malvidin 3 glucoside	−0.55	−0.53	−0.94
Malvidin 3-(6′acetyl)-glucoside	−0.32	−0.35	0.41
Pet. 3-(6″coumaroyl)-glucoside	0.47	0.59	0.23
Mal. 3-(6″coumaroyl)-glucoside	−0.24	−0.11	−0.18

## Data Availability

Data are contained within the article.
